# Healthy Aging Through the Senior Years (HATS) Study: A longitudinal cohort study of brain health and dementia among Black older adults in the United States

**DOI:** 10.1002/alz.087005

**Published:** 2025-01-09

**Authors:** Kerry M. Sheets, David S. Knopman, Ronald C. Petersen, Prashanthi Vemuri, Clarence Jones, Anne Murray

**Affiliations:** ^1^ Berman Center for Outcomes and Clinical Research, Hennepin Healthcare Research Institute, Minneapolis, MN USA; ^2^ Hennepin Healthcare, Minneapolis, MN USA; ^3^ University of Minnesota, Minneapolis, MN USA; ^4^ Mayo Clinic, Rochester, MN USA; ^5^ Hue‐MAN Partnership, Minneapolis, MN USA

## Abstract

**Background:**

The higher prevalence and incidence of later life dementia among older Black Americans compared to older White Americans is incompletely understood and understudied. HATS is designed as a companion to the Mayo Clinic Study of Aging (MCSA) with a focus on identification of modifiable cardiovascular contributions to brain health among Black older adults living in urban areas in the upper Midwest.

**Method:**

HATS is enrolling 300 U.S. Black Minnesotans from the Twin Cities area aged 55 to 90 years without advanced dementia. Enrollment started 4/2023 and is expected to be complete by the end of 2025. Recruitment is primarily community‐based, led by two community engagement partners, the Linc and the Hue‐MAN Partnership. Electronic health record‐based recruitment will begin in 2024. Data collection includes medical comorbidities, structural and social determinants of health, cardiovascular health, functional status, physical performance, specific and non‐specific blood‐based biomarkers of Alzheimer’s disease, and comprehensive assessment of cognitive and neuropsychiatric status. Brain MRIs will be obtained on a subset of participants. A core component of HATS is distribution of meaningful health information back to participants and their community, guided by input from the HATS community engagement partners. The HATS cohort will serve as an initial effort to develop age and education adjusted normative cognitive test data for urban Black older adults in the upper Midwest.

**Result:**

As of January 22 2024, HATS has enrolled 40 participants with a mean age of 70 years (range 51‐90) and mean education of 15 years. Mean informant‐reported Everyday Cognition scale‐12 score is 1.1 (range 1‐1.7), mean Short Test of Mental Status (STMS) score is 31 (range 24‐37), and CDR sum of boxes scores range from 0‐4 consistent with enrollment of participants with normal and mildly impaired cognition.

**Conclusion:**

HATS is a novel longitudinal cohort study that is well‐positioned to advance understanding of risk factors for dementia among Black Americans living in urban areas in the upper Midwest. Recruitment is ongoing with a focus on enrolling older adults with a wide range of cognitive function. Ongoing community engagement is a central feature of HATS.

